# Diffuse Large B-Cell Lymphoma Involvement in Two Different Localizations: A Case Report

**DOI:** 10.1055/s-0039-1678709

**Published:** 2019-03-04

**Authors:** Nilufer Bulut, Hılal Serap Arslan, Ipek Yıldız Ozaydın

**Affiliations:** 1 Department of Medical Oncology, Kanuni Sultan Suleyman Education and Research Hospital, Istanbul, Turkey; 2 Department of Pathology, Kanuni Sultan Suleyman Education and Research Hospital, Istanbul, Turkey

**Keywords:** B-cell lymphoma, skin, uterine, treatment

## Abstract

The genital system and skin involvement of diffuse B-cell lymphomas are quite rare. The appearance of these rare types in the same patient and the same period makes the treatment of the disease difficult. But both types respond well to anthracyclines and immunotherapies. A 74-year-old woman was treated with R-CHOP (Rituximab, cyclophosphamide, doxorubicine, vincristine, prednisolone) without surgery and/or radiotherapy, and no recurrence at 2 years follow-up. Despite the poor prognosis of these types of lymphomas, treatment responses are quite good as they are in other subtypes.

Malignant lymphoma accounts for 0.05% of uterine malignancies in women . 1 Uterine cervix is frequently affected, whereas endometrial corpus involvement is rare. Therefore, there is no standard treatment for malignant lymphoma, but treatment options include surgery, chemotherapy, or combination of surgery and immunotherapy. Primary cutaneous large cell lymphoma, leg type, is a rare and fast-growing neoplasm that accounts for 5 to 10% of all cutaneous B-cell lymphomas. 2 It is often located on the lower extremity. Immunochemotherapy and/or involved radiotherapy (RT) are used for the treatment.

We report the case of a woman who developed diffuse large B-cell lymphoma (DLBCL) on endometrium and on skin of lower extremity. Patient received six courses of R-CHOP and complete remission was achieved. It was observed that complete response was achieved for two fast-growing lymphomas.

## Case Report

**Fig. 1:**
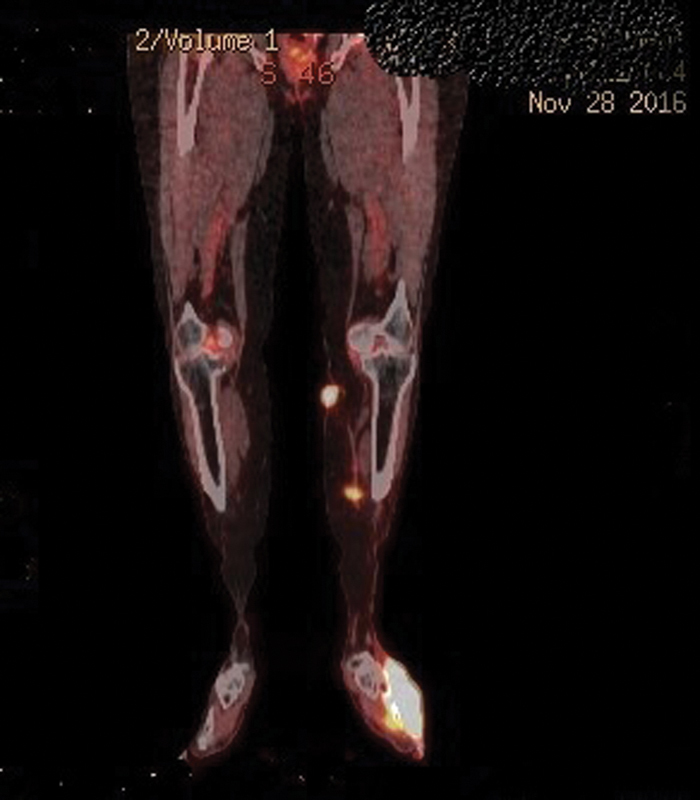
FDG-PET/CT showed a primary lesion at the dorsum of the left foot (SUV: 13.6), extensor muscles, and subcutaneous fat tissue. CT, computed tomography; FDG, fluorodeoxyglucose; PET, positron emission tomography.

**Fig. 2:**
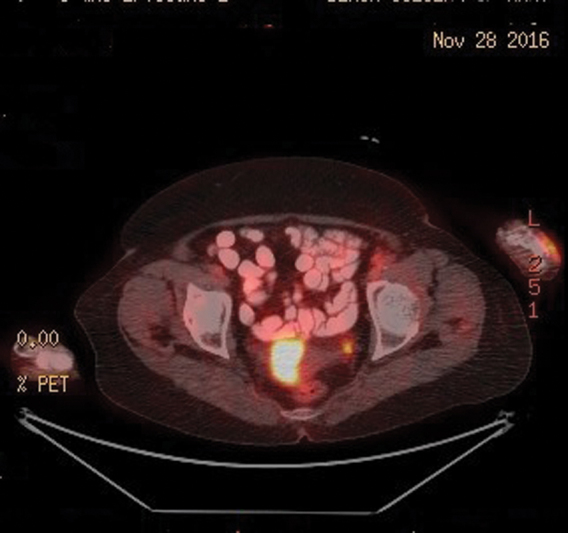
FDG-PET/CT showed a mass filling the uterine cavity and metastatic lymph node (SUV: 9.1). CT, computed tomography; FDG, fluorodeoxyglucose; PET, positron emission tomography.

**Fig. 3:**
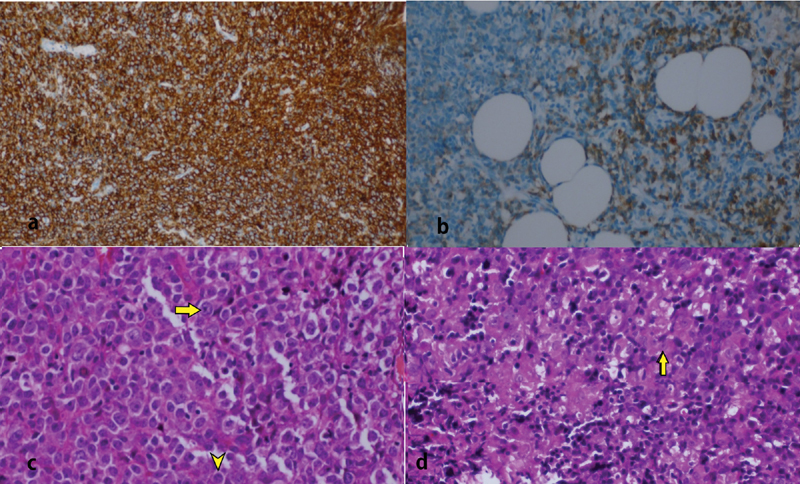
Immunohistochemical staining revealed positive (a) CD20, (b) CD79a, (c) above marked immunoblast, below centroblast cell, bcl-6 (+) on uterine specimen (HE ×400), and (d) immunoblast cell in cutaneous biopsy (HE ×400). HE, hematoxylin and eosin.

**Fig. 4:**
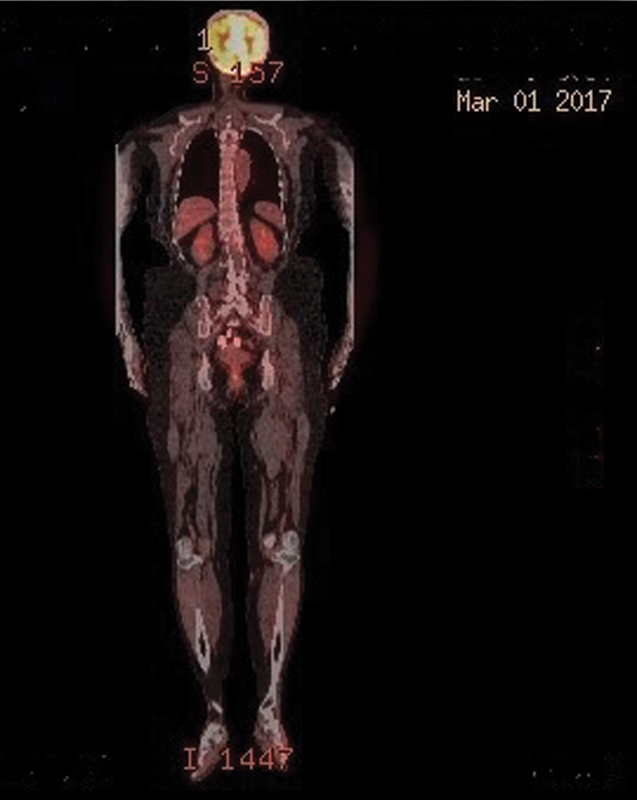
Complete remission after immunochemotherapy was showed on uterine and crural region on FDG-PET/CT (SUV: 2). CT, computed tomography; FDG, fluorodeoxyglucose; PET, positron emission tomography.

A 74-year-old postmenopausal female presented to dermatology outpatient clinic with the complaint of a mass on dorsum of the left foot and a mass on the middle part of the medial surface of the left lower extremity for a month. A tru-cut biopsy was obtained from malignant necrotic ecchymosis under antibiotic prophylaxis. Magnetic resonance imaging (MRI) of the pelvic and MRI of the lower extremity were performed. The results showed a mass obliterating the endometrium and a pathologic lymphadenopathy, measuring 30 × 15 mm, on the left inguinal region. Vaginal ultrasound was performed and adnexal atrophy and cysts in uterus were detected. Dilatation and curettage was performed on endometrium and patient was diagnosed with high-grade cancer. MRI of the lower extremity showed a heterogeneous mass, which is measuring 56 × 40 mm and is infiltrating proximal parts of third and fourth metatarsal bones and muscle and fat tissue on dorsal part of left foot. Positron emission tomography–computed tomography (PET-CT) was performed for stating. The results demonstrated a lesion, measuring 10.5 × 6.5 × 5.5 cm, extending from skin to extensor muscles and infiltrating proximal fourth and fifth phalanges in intermetatarsal spaces on plantar surface of the foot (SUVmax: 13.6). A primary lesion, measuring 25 mm in diameter, that is extending from medial calcaneus to subcutaneous tissue on proximal and one-third part of the middle part of left lower extremity (SUVmax: 26.5) and a second primary mass, measuring 45 × 28 mm, that is filling corpus of the uterus (SUVmax: 45.8) are noted. A metastatic lymph node, measuring 2 cm in diameter, is noted (SUVmax: 9.1) ( Figs. 1 and 2 ). The results of the biopsy were evaluated and patient was diagnosed with high-grade DLBCL. The both tumors histopathological features were similar. This was in accordance with germinal centered high-grade non-Burkitt DLBCL. DLBCL-leg was diagnosed when MUM1 (+), CD20 (+), CD3 (−), CD5 (−), CD38 focal (+), CD79a (+), c-myc 20 to 30% (+), bcl-2 (+), bcl-6 (+), and CD10 (−) were evaluated with clinical findings ( Fig. 3a – d ).The uterine tumor was diagnosed as stage IEA DLBCL according to the Lugano modification of Ann Arbor classification, the other tumor was diagnosed as T2aN1M0 according to WHO-EORTC classification and R-CHOP chemotherapy was initiated. After three courses of R-CHOP, PET-CT was performed. The scan showed almost complete regression in mass in uterus; complete regression in lesion on dorsum of the left foot and partial regression in anterior lesion of left lower extremity. After six courses of chemotherapy, PET-CT was performed and it demonstrated a residual mass extending to subcutaneous tissue and muscles of dorsum of left foot (SUVmax: 2.0) ( Fig. 4 ). A skin biopsy was performed. The results showed no cutaneous lymphoma, but atrophy of the dermis was detected. No residual mass was noted histopathologically; therefore, salvage RT was not planned.

## Discussion

Primary lymphoma of the endometrium is often detected by vaginal hemorrhage in elderly postmenopausal women without constitutional symptoms. Patients are treated with radical hysterectomy and lymph node dissection, followed by R-CHOP therapy. The 5-year overall survival rate is 39.3 %. In some cases, the complete remission rate of immunochemotherapy without surgery is 75%. 3

Primary cutaneous B-cell lymphoma is often observed in elderly women. Lesions are present on head, neck, trunk, or lower extremity. Regardless of skin localization, MUM1/IRF4, bcl-2, and FOX-P1 are strongly expressed. 4 This immunophenotype may be negative in 10% of cases. The 5-year overall survival rate is 40 to 55%. 5 Multiple skin lesions, high expressions of MUM1, FOX-P1, and bcl-2 positivity are poor prognostic. 5 R-CHOP chemotherapy or involved-field RT is used for the treatment.

Two rare types of lymphoma detected in the same patient were treated with R-CHOP chemotherapy. A very good partial response was achieved at the end of three courses, and complete remission was achieved at the end of six courses. Considering that there was no residual disease and that sequelae effects of RT administered to the foot would adversely affect the quality of life, involved-field RT was not administered to the patient. In the literature, cutaneous or extracutaneous relapses have been reported frequently, despite good response to immunochemotherapy. In this case, there was no recurrence of both endometrium and skin for 2 years. Immunologically, B surface markers such as CD20, CD79a, Pax-5, and bcl-2 are frequently positive in diffuse cutaneous B-cell lymphomas. 6 While MUM-1/IRF4, FOX-P1, and P63 were strongly positive in cutaneous leg-type lymphomas, other markers such as CD3 (−), CD5 (+/−), CD20 (+), CD79a (+), bcl-6 (+), and K _i_ -67 in endometrial involvement were 60% positive. 7 Complete response was obtained with immunotherapy and neither surgery nor RT was performed. 7 Immunologic markers have an indefinite prognostic relevance, with a response rate of 92% for anthracycline-containing treatments. 6

## Conclusion

It is very rare to detect uterine corpus and cutaneous B-cell lymphoma, leg type, simultaneously in the same patient. During diagnosis, PET-CT and MRI examinations demonstrated involvements other than primary focus. Although both foci are fast growing histologically, it was observed that complete remission could be achieved with immunochemotherapy.
